# Interactions of factors in self-injuries among enrolled students: a network approach

**DOI:** 10.1186/s12991-025-00570-0

**Published:** 2025-05-16

**Authors:** Yu-Min Zhang, Xiao-Mei Jiang, Ya Xie, Nan Lang, Min-Lu Liang, Pei Zhang, Li-Chen OuYang, Zhang-Wei Lv, Cong-Wei Liu, Li-Ping Zhang, Chun Wang

**Affiliations:** 1 The Affiliated Brain Hospital of Nanjing Medical University, 264 Guangzhou Rd, Nanjing, Jiangsu China; 2 Gansu Gem Flower Hospital, Lanzhou, China; 3 School of Psychology, Nanjing Normal University, Nanjing, Jiangsu China

**Keywords:** Non-suicidal Self-injury, Suicidal ideation, Suicide attempt, Enrolled students

## Abstract

**Purpose:**

Suicidal and non-suicidal self-injuries are types of self-directed violence that can become complex health issues. This study assessed how and to what degree the factors of self-injuries are interrelated among enrolled students.

**Methods:**

A total of 1481 students were recruited from college and middle or secondary schools, and 1465 (98.92%) subjects comprised the final sample. Mixed graphical models were used to establish network structures. Also explore their shortest paths and conduct a regression analysis.

**Results:**

Of the 1465 students, we observed intersections that connected the cluster of early experiences and psychiatric/psychological using network analysis. Shortest paths analysis and regression analysis suggest that symptoms of schizoid (edge-weights = 0.336, OR = 2.79, *p* < 0.01) and narcissistic (edge-weights=-0.177, OR = 0.35, *p* < 0.05) personality disorders (PD), acceptance (edge-weights = 0.470, OR = 12.80, *p* < 0.01) and positive refocusing (edge-weights=-0.171, OR = 0.12, *p* < 0.05) strategies of emotion-regulation, mindfulness awareness (edge-weights=-0.263, OR = 0.24, *p* < 0.05), and emotional-neglect in childhood (edge-weights = 0.239, OR = 5.54, *p* < 0.05) were found with non-suicidal self-injury (NSSI). Symptoms of anxiety (edge-weights = 0.280, OR = 2.00, *p* < 0.01) and avoidant-PD (edge-weights = 0.229, OR = 1.75, *p* < 0.01) were associated with suicidal ideation, and symptoms of borderline-PD (edge-weights = 0.432, OR = 5.38, *p* < 0.05) and mindfulness awareness (edge-weights=-0.180, OR = 0.28, *p* < 0.05) were associated with suicide attempt.

**Conclusions:**

Relying exclusively on acceptance strategy may constitute an avoidance pattern, impeding the ability to confront emotional distress. Clinical intervention aimed at repairing father-child relationship may be helpful to recover from emotional trauma and improve current symptoms and self-injuries.

**Supplementary Information:**

The online version contains supplementary material available at 10.1186/s12991-025-00570-0.

## Introduction

Suicidal and non-suicidal self-injuries (hereinafter, self-injuries) are types of self-directed violence that can become complex health issues. In a broad sense, self-injurious behaviors (SIBs) encompass all intentional behaviors performed with the knowledge that they can or will cause injury to oneself to some degree [1]. Intent to die can be used to distinguish suicidal from non-suicidal self-directed violence [2]. Non-suicidal self-injury (NSSI) is defined as acts of self-oriented, deliberate destruction or alteration of body tissue without suicidal intent [1]. The prevalence of NSSI for students worldwide is about 13-45% [1]. Suicidal behavior is divided into suicidal ideation (SI), suicide attempt (SA) and suicide committed [2]. SA is defined as a nonfatal, self-directed, potentially injurious behavior with an intent to die as a result of the behavior even if the behavior does not result in injury, and SI consists of suicidal thoughts, considerations, or plans [2]. Lifetime global prevalence rates of SI and SA are approximately 9.2% and 2.7%, respectively [2].

It is generally believed that self-injuries result from the interaction of biology, psychology, and environment. From a developmental perspective, distal factors (such as early-life adversity) are proposed to increase the likelihood of mediating factors (such as psychological vulnerabilities) that set the stage for proximal or precipitating factors (such as mental disorder symptoms), which then lead to problematic reactions to stress [1, 3]. Several studies have explored the relationship between distal factors and increased risk of self-injuries. Childhood trauma experiences, including abuse and neglect, have long been recognized as having significant effects on self-injuries [4, 5]. Youth with histories of NSSI were found to be more likely to have suffered from any kind of such maltreatment [5]. Broader family-related factors also contribute to the self-injurious risk. For instance, family conflict has been associated with an increased likelihood of both suicidal and non-suicidal self-injuries [6]. In this context, negative parental behaviors, encompassing both maternal and paternal styles, may contribute to shaping psychological vulnerability in adolescents, while positive parental involvement can serve as a protective buffer [7]. Additional studies have explored how mediating factors contribute to increased risk of these behaviors. Certain vulnerabilities increase the likelihood of self-injurious behavior because they map onto the associated functions of this behavior [1]. Psychological factors were considered more salient in adolescents and young adults with suicidality, which explains why susceptible individuals who display altered cognitive patterns are more likely to have SI after a stressful experience [3, 8]. This also sheds lights on the relationship between high level of impulsive-aggressive traits and high rate of SA among youth [8]. Proximal predictors have been implicated as the precipitants of self-injuries. Previous research suggested that a prior history of SIBs is one of the strongest predictors of SA both cross-sectionally and longitudinally [3]. Psychopathology is another proximal predictor in the risk of self-injuries. Depression was found to differentiate SIBs with or without ideation [9]. Likewise, NSSI and some psychiatric symptoms (e.g., major depressive disorder, MDD) confer risk for SI through partially similar pathways [10].

Since the associating factors may act together in forming and maintaining self-injuries and are affected reciprocally by the behaviors, we used multiple analysis techniques to probe these relationships. Network theory produces a visual guide to estimate relevance underlying multivariate data, as it allows all the interactions to be considered in one study [11]. Gaussian graphical model (GGM) was first proposed to use broadly in contemporary exploratory graph analysis [11]. Lately, mixed graphical model (MGM) has emerged, which allows combining an arbitrary set of conditional univariate members of the exponential family in a joint distribution, instead of GGM that only estimates the normal distribution [11–13].

The purpose of this study was to assess how and to what degree the associating factors of suicidal and non-suicidal self-injuries are interrelated within the self-injury network among students and which factors play a central role.

## Methods

### Participants and procedures

The inclusion criterion for this study was being an enrolled student from colleges and middle or secondary schools between July 2020 and April 2021, while the exclusion criterion encompassed individuals not currently enrolled in school (e.g., academic leave). A total of 1481 students were initially recruited through an online platform. Of these, 16 participants who submitted incomplete responses to the questionnaires were excluded from the analysis. As a result, the final sample included 1465 students.

### Measures

Overall, 3 self-injurious variables and 50 associating variables were assessed through 16 self-reporting scales. We divided these 50 outcomes into proximal, mediating, and distal factors. Table S1 at the Supplementary Information describes the variables for the network structure including respective recording, scoring and abbreviations.

#### Suicidal and non-suicidal self-injuries

The students were asked to report their NSSI frequency over the past year using an excerpt of Ottawa/Queen’s Self-Injury Questionnaire (OSI) [14]. We recoded these frequencies as “no NSSI” and “NSSI” (at least once), with the *NSSI* node (node 51) labeled in the network structure.

The students also reported their suicidality (including SI and SA) using a simple version of Columbia Suicide Severity Rating Scale (C-SSRS) [15], which consists of 5-levels suicidal thoughts and 4-types suicidal behaviors. We re-encoded the responses as “no SI” and “SI” (with arbitrary suicidal thoughts), and “no SA” and “SA” (with arbitrary suicidal behaviors), with the *SI* and *SA* nodes (nodes 52 and 53) labeled in the network structure.

#### Proximal factors

Fourteen psychological variables, including various emotional and personality problems, were considered proximal factors. Emotion symptoms consisted of depression, mania or hypomania and anxiety. Personality symptoms were taken from ten types of personality disorders (PD) as well as borderline personality disorder.

The depression and anxiety, labeled as *DepA* and *AnxA* (nodes 1 and 3), were assessed with Zung’s Self-rating Depression Scale (SDS) [16] and Zung’s Self-rating Anxiety Scale (SAS) [17]. The mania or hypomania, labeled as *ManA* (node 2), was assessed with the Chinese version of the Mood Disorder Questionnaire (MDQ) [18]. Cronbach’s alphas were 0.76 for both SDS and SAS, and 0.73 for MDQ. To screen symptoms of PD, the Short Version of the Borderline Symptom List (BSL-23; 23-item) and Personality Diagnostic Questionnaire 4th edition (PDQ-4) were used. BSL-23 is a well-established instrument that measures borderline-typical symptomatology [19], which is labeled as *BorS* node (node 4) in the network structure. PDQ-4 yielded personality diagnoses for ten officially recognized PDs [20], including paranoid PD (*PPD*, node 5), schizoid PD (*ScPD*, node 6), schizotypal PD (*SctPD*, node 7), antisocial PD (*AnPD*, node 8), borderline PD (*BPD*, node 9), histrionic PD (*HPD*, node 10), narcissistic PD (*NPD*, node 11), avoidant PD (*AvPD*, node 12), dependent PD (*DPD*, node 13), and obsessive-compulsive PD (*OPD*, node 14).

Cronbach’s alpha was 0.97 for BSL-23, and 0.95 for PDQ-4 total scale, with a mean of 0.69 for subscales ranging from 0.63 to 0.77.

#### Mediating factors

Psychological variables related to emotion, cognition and disposition were considered mediating factors and were evaluated with seven instruments.

Wong-Law Emotional Intelligence Scale (WLEIS) [21] and Toronto Alexithymia Scale (TAS) [22] were used to assess emotional intelligence (*EI*, node 15) and severity of alexithymia (*Alx*, node 16), respectively. Cognitive Emotion Regulation Questionnaire (CERQ) [23] were used to assess nine reference strategies after having experienced negative life events, including positive refocusing (*Cprf*, node 17), refocus on planning (*Crop*, node 18), positive reappraisal (*Cpra*, node 19), acceptance (*Cacc*, node 20), putting into perspective (*Cpip*, node 21), self-blame (*Csb*, node 22), rumination (*Crum*, node 23), catastrophizing (*Ccat*, node 24), blaming others (*Cbo*, node 25). Impulsive traits (*Ips*, node 28) was evaluated using Barratt Impulsiveness Scale (BIS-11) [24]. Mindfulness attention awareness (*MAA*, node 26), a core characteristic of dispositional mindfulness, was evaluated using Mindfulness Attention Awareness Scale (MAAS) [25]. Psychological resilience (*PR*, node 27), a quality that enables individuals to thrive in the face of adversity, was evaluated using Connor-Davidson Resilience Scale (CD-RISC) [26]. Coping Style Questionnaire (CSQ) was used to evaluate six coping styles, including avoiding (*CSav*, node 29), fantasizing (*CSfa*, node 30), self-blaming (*CSsb*, node 31), seeking help (*CSsh*, node 32), rationalizing (*CSra*, node 33), problem solving (*CSps*, node 34).

Cronbach’s alpha for the scales ranged from 0.68 to 0.96, with a mean of 0.90.

#### Distal factors

Sixteen distal factors related to upbringing experiences (childhood trauma and growing environment) were divided into nodes and were evaluated using two instruments.

Nodes from 46 to 50 correspond to five types of trauma experiences during childhood: emotional abuse (*CTea*), physical abuse (*CTpa*), sexual abuse (*CTsa*), emotional neglect (*CTen*), and physical neglect (*CTpn*), as measured by Childhood Trauma Questionnaire 28-item short form (CTQ-SF) [27]. The Chinese version of the Swedish EMBU inventory (C-EMBU) [28] were used to measure six parental rearing patterns. Nodes 35 to 40 correspond to paternal patterns: affectionate and tolerant (*Fat*), abusive and punitive (*Fap*), overinvolved (*Fo*), favored subject (*Ffs*), rejecting and shaming (*Frs*), and overprotective (*Fop*), while 41 to 45 represent corresponding maternal patterns (*Mat*, *Map*, *Mo*, *Mfs*, and *Mrs*).

Cronbach’s alpha was 0.80 for CTQ-SF, and 0.97 for C-EMBU.

### Data analysis

Data processing was carried out using SPSS (Version 23.0) and statistical analyses were performed using R Studio (Version 1.4.1717). The network analysis was performed using packages *qgraph* (Version 1.9), *mgm* (Version 1.2–12) and *bootnet* (Version 1.5). Logistic regression analysis was conducted using packages *vif* (Version 3.0–12) and *glm* (Version 4.1.2).

#### Network analysis

First, we constructed two separate networks using MGM: a factor network (including fifty associating factors) and a self-injury network (including the factors and self-injuries). The Type I error rate was controlled by L1-penalty regularization with 10-fold cross-validation. Nodes of self-injuries are colored in red, and the associating factors are in green, blue, and yellow, corresponding to proximal, mediating, and distal factors, respectively. The rings display nodewise errors from the model; the blue rings indicate the proportion of variance explained (R2) by neighboring nodes for the Gaussian variables, and the red rings indicate the proportion of correct classification (CC) of the categorical nodes [13]. Connections between nodes represent the strength of the statistical association that remains after controlling for other nodes [13]. We excluded values below 0.10 in order to obtain a clearer and simpler graphic. Edges colored in green indicate positive statistical associations, and red indicates negative statistical associations. The stronger a correlation, the more saturated and wider the edge.

Secondly, centrality metrics indicating the overall connectivity of variables was computed. Strength centrality (the absolute sum of the edge weights connected to a node) investigates how strongly a node is directly connected to others [12]. Closeness centrality (the inverse of the sum of the shortest path lengths between one node with all other nodes) quantifies how strongly a node is indirectly connected to others [12, 29]. Betweenness centrality (the number of shortest paths connecting any two nodes) indicates how many of the shortest paths between two nodes go through the node [12, 29]. To check the robustness of the centralities, we conducted a bootstrap procedure using 2500 bootstraps (α = 0.05), with a correlation stability coefficient (CS-coef) based on subset bootstraps. The CS-coef represents the proportion of the sample that can be removed while maintaining a correlation of *r* = 0.7 between the original centrality indices and those from case-dropped bootstraps. The CS-coef should not be below 0.25, and preferably above 0.5 to interpret differences [12].

Lastly, we estimated shortest paths (the paths with the maximum product of weights) to explore the direct connectivity patterns between factors and each type of self-injury separately.

#### Logistic regression analysis

Binomial logistic regression was used to verify the associations with self-injury. Variance inflation factors (VIF) ranged from 1.15 to 8.38, which commonly indicating no serious multicollinearity (VIF < 10). Thus, we included all 50 variables and calculated odd ratios (OR) and their 95% confidence intervals (95%CI) for self-injuries separately.

## Results

The sample was 35.36% male (*n* = 518) and 64.64% female (*n* = 947), with an age range from 12 to 25 (*M* = 18.02, *SD* = 3.00). Among the participants, 38.29% (*n* = 551) were receiving secondary education (12-year compulsory education), with an average age of 15.32 (*SD* = 1.70). 61.71% (*n* = 888) were receiving tertiary education, with an average age of 19.62 (*SD* = 2.36).

### Network analysis

#### Network constructions and centralities

**Fig. 1:**
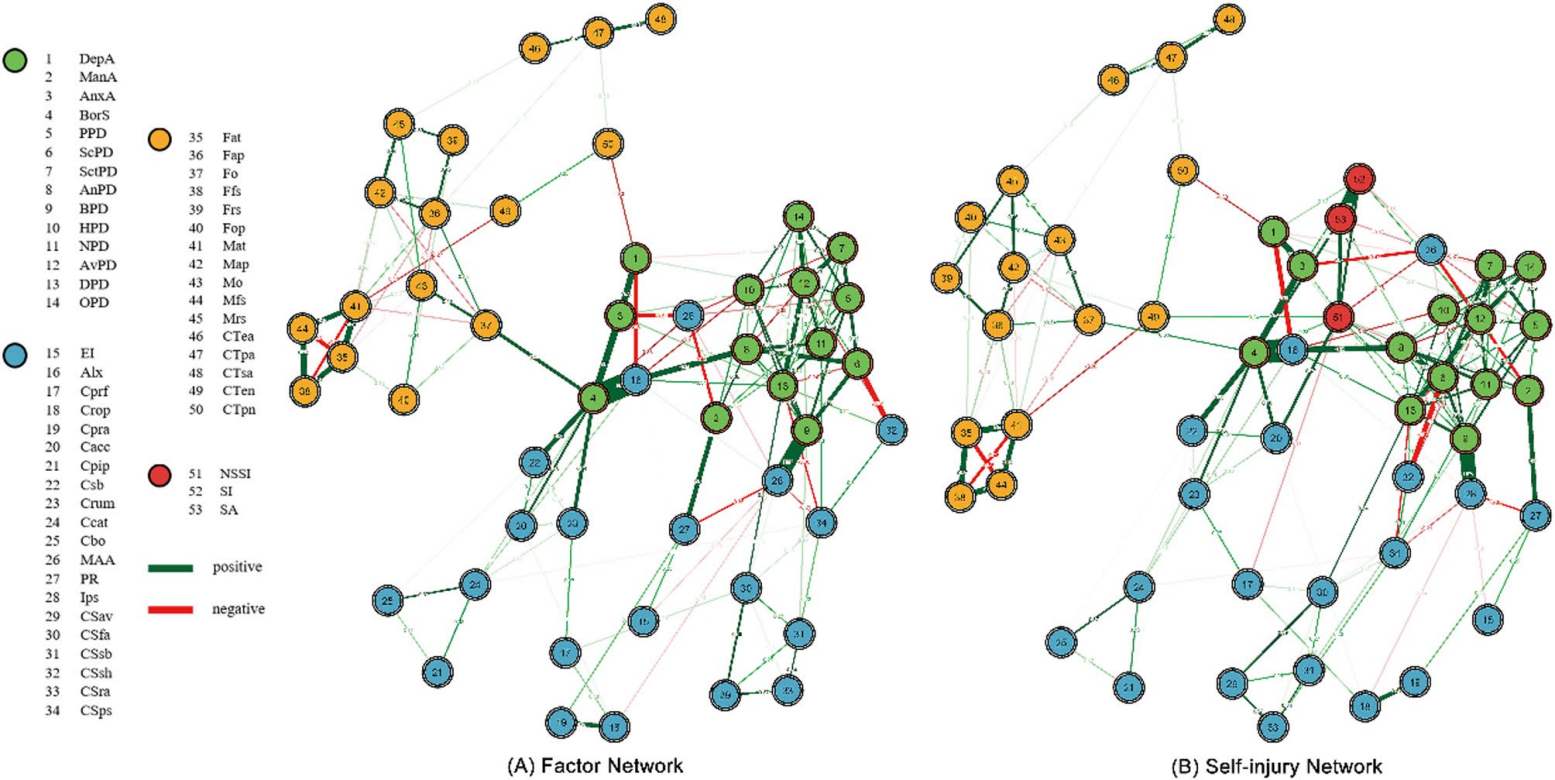
Estimated factor network structure (mixed graphical models, cutoff = 0.10) of the students (**A**) Factor Network including 50 associating factors, and (**B**) Self-injury Network including 50 associating factors with 3 self-injuries

Figure 1 presents network structures of factor network (Fig. 1A), with 222 non-zero edges out of 1225 possible edges due to least absolute shrinkage and selection operator (LASSO) estimation, and self-injury network (Fig. 1B), with 239 non-zero edges out of 1378 possible edges. We visually identified a cluster of distal factors (see yellow nodes in Fig. 1A) that connected to proximal and mediating factors (see green and blue nodes in Fig. 1A) mainly across two pairs of interactions (*DepA*- *CTpn* and *BorS*-*Fo)*. After adding the nodes self-injuries, we observed an extra interaction (*NSSI-CTen*) connecting the distal cluster (see yellow nodes of Fig. 1B) and the others (see red, green and blue nodes in Fig. 1B). In self-injury network, *Cacc* had the strongest independent association with *NSSI* (edge wights = 0.470), *AnxA* with *SI* (edge wights = 0.280), and *BorS* with *SA* (edge wights = 0.432).

**Fig. 2:**
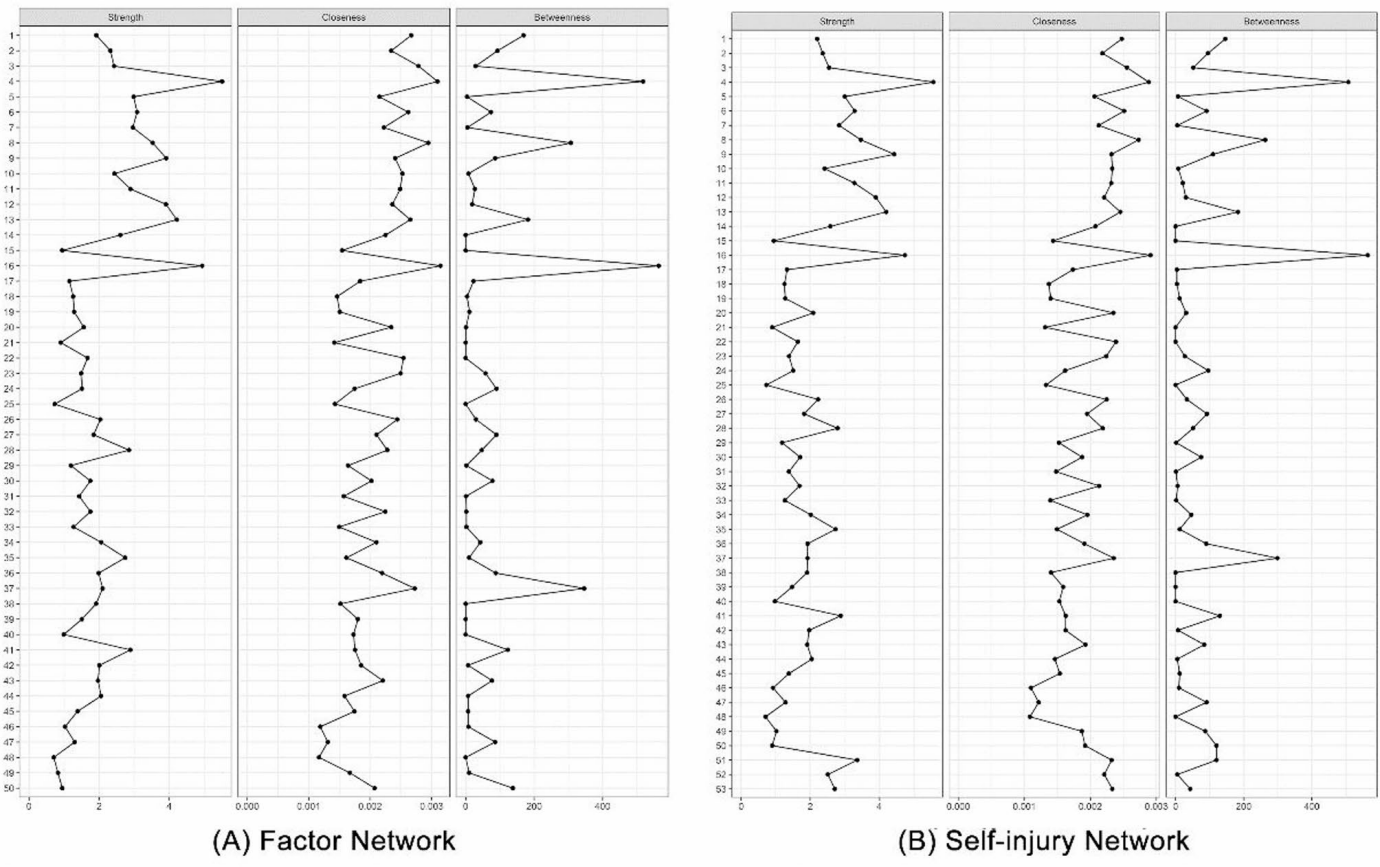
Centralities of (**A**)Factor Network, and (**B**) Self-injury Network. The nodes are numbered as follows: node number from 1 to 14 are proximal factors; node number from 15 to 34 are mediating influences; node number from 35 to 50 are distal factors, node number from 51 to 53 are self-injuries

Figure 2 displays the centrality plots of factor network (Fig. 2A) and self-injury network (Fig. 2B). In factor network, *BorS* (strength = 5.510–5.570, closeness = 0.003, betweenness = 507–518) and *Alx* (strength = 4.740–4.940, closeness = 0.003, betweenness = 564) were the top three centrality, but *CTsa* (strength = 0.704–0.705, closeness = 0.001, betweenness = 0) and *Cbo* (strength = 0.729–0.732, closeness = 0.001, betweenness = 0) connected weakly with others. However, some variations occurred in the self-injury network. Centralities of *BPD* (strength = 4.430), *Cacc* (strength = 2.090) and *CTen* (betweenness = 87) increased after adding self-injuries (*BPD*, strength = 3.910; *Cacc*, strength = 1.560; *CTen*, betweenness = 10), while *AnPD* (betweenness = 307) and *Fo* (betweenness = 346) centralities lowered (*AnPD*, betweenness = 263; *Fo*, betweenness = 299).

#### Shortest paths to self-injuries

**Fig. 3:**
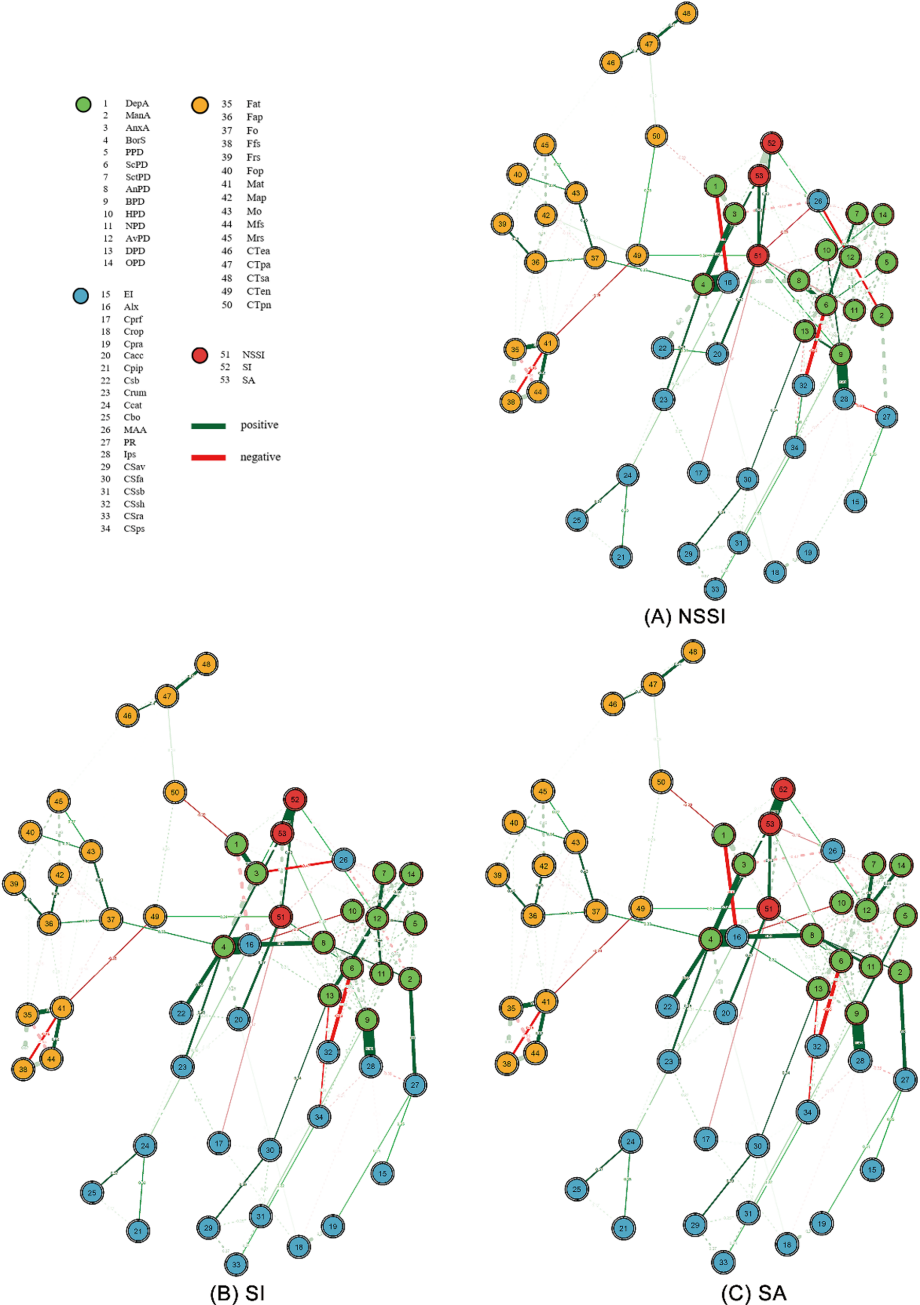
Shortest paths from associating factors to (**A**) NSSI, (**B**) SI, and (**C**) SA. Solid lines indicate shortest paths within the network, while dashed ones indicate background connections that are less relevant when investigating shortest path

Figure 3 depicts the shortest paths from associating factors to each self-injury. Direct connections generally involving proximal, mediating and distal factors were found with *NSSI* (Fig. 3A); *ScPD* (edge-weights = 0.336), *BPD* (edge-weights = 0.246), *NPD* (edge-weights=-0.177), *Cacc* (edge-weights = 0.470), *Cprf* (edge-weights=-0.171), *MAA* (edge-weights=-0.171) and *CTen* (edge-weights = 0.239). The shortest paths with positive connections focused on proximal factors to suicidality; *AnxA* (edge-weights = 0.280) and *AvPD* (edge-weights = 0.229) were associated with *SI* (Fig. 3B), and *BorS* (edge-weights = 0.432) and *BPD* (edge-weights = 0.188) were associated with *SA* (Fig. 3C). Additionally, a negative connection was found between *MAA* (edge-weights= -0.180) and *SA* (Fig. 3C).

#### Network stability

Figure S1 at the Supplementary Information presents the accuracy and stability of the network and includes bootstrap samples for the factor network (Figure S1A) and self-injury network (Figure S1B). As participants were removed, the strengths stabilized (CS-coef = 0.595) in both networks. Moreover, closeness was higher in the self-injury network (CS-coef = 0.283) than in the factor network (CS-coef = 0.128). The CS-coef of betweenness varied from 0 (self-injury network) to 0.50 (factor network). Therefore, interpretations for the differences between nodes regarding centralities of closeness and betweenness should be with caution.

### Logistic regression analysis

Table 1 displays the results of logistic regression analysis for associating factors and self-injuries. The connections involving NSSI generally coincided with the shortest paths. Besides, Cpip (OR = 6.75; 95%CI, 1.18–38.69; *p* < 0.05) was found to have a positive influence on NSSI. No correlation between BPD (OR = 1.97; 95%CI, 0.96–4.01; *p* = 0.063) and NSSI was found. Logistic regression showed that DepA (OR = 1.48; 95%CI, 1.10–2.01; *p* < 0.05), Cpra (OR = 4.15; 95%CI, 1.17–14.73; *p* < 0.05), Crum (OR = 2.56; 95%CI, 1.00-6.65; *p* < 0.05), Cbo (OR = 2.78; 95%CI, 1.12–6.91; *p* < 0.05), Cea (OR = 32.40; 95%CI, 7.53-139.11; *p* < 0.001) and DPD (OR = 0.57; 95%CI, 0.34–0.94; *p* < 0.05) had significant effects on SI. Cpra (OR = 0.08; 95%CI, 0.01–0.53; *p* < 0.01), Frs (OR = 0.10; 95%CI, 0.01–0.95; *p* < 0.05) and Fap (OR = 49.70; 95%CI, 4.50-548.93; *p* < 0.001) had significant influences on SA in regression analysis, but BPD (OR = 1.54; 95%CI, 0.85–2.80; *p* = 0.157) did not.

**Table 1:** Logistic regression analysis of influencing factors on suicidal and non-suicidal self-injuries among enrolled students

VARIABLES	NSSI			SI			SA	
	OR	95%CI		OR	95%CI		OR	95%CI
DepA (ref: 0)	1.49	[0.85–2.61]		1.48	[1.10–2.01] ^*^		0.83	[0.53–1.31]
ManA (ref: 0)	1.90	[0.95–3.77]		0.80	[0.50–1.27]		0.89	[0.46–1.73]
Anxa (ref: 0)	0.93	[0.50–1.74]		2.00	[1.31–3.04] ^**^		1.45	[0.87–2.43]
BorS (ref: 0)	1.85	[0.46–7.50]		0.26	[0.06–1.19]		5.38	[1.27–22.74] ^*^
PPD (ref: 0)	0.75	[0.40–1.40]		0.91	[0.61–1.36]		1.20	[0.71–2.03]
scPD (ref: 0)	2.79	[1.41–5.54]^**^		1.01	[0.60–1.69]		1.26	[0.68–2.33]
SctPD (ref: 0)	1.05	[0.53–2.08]		0.78	[0.49–1.25]		1.53	[0.86–2.71]
AnPD (ref: 0)	0.77	[0.34–1.76]		1.40	[0.76–2.58]		1.28	[0.64–2.56]
BPD (ref: 0)	1.97	[0.96–4.01]		1.18	[0.72–1.93]		1.54	[0.85–2.80]
HPD (ref: 0)	1.22	[0.67–2.23]		0.71	[0.48–1.05]		1.37	[0.82–2.31]
NPD (ref: 0)	0.35	[0.15–0.82]^*^		1.15	[0.67–1.97]		0.61	[0.31–1.24]
AvPD (ref: 0)	1.49	[0.82–2.72]		1.75	[1.25–2.46] ^**^		0.84	[0.51–1.40]
DPD (ref: 0)	1.05	[0.49–2.27]		0.57	[0.34–0.94] ^*^		1.42	[0.74–2.71]
OPD (ref: 0)	0.80	[0.45–1.42]		1.07	[0.76–1.50]		1.07	[0.67–1.72]
EI	5.14	[0.64–41.36]		1.17	[0.39–3.51]		0.19	[0.03–1.09]
Alx	0.57	[0.06–5.11]		1.98	[0.60–6.56]		0.79	[0.12–5.39]
Cprf	0.12	[0.02–0.62]^*^		1.02	[0.39–2.70]		1.63	[0.37–7.09]
Crop	1.96	[0.22–17.61]		0.96	[0.27–3.38]		3.98	[0.64–24.82]
Cpra	2.37	[0.29–19.10]		4.15	[1.17–14.73] ^*^		0.08	[0.01–0.53] ^**^
Cacc	12.80	[2.27–72.09] ^**^		0.56	[0.21–1.54]		1.24	[0.28–5.54]
Cpip	6.75	[1.18–38.69] ^*^		0.48	[0.18–1.32]		1.18	[0.25–5.58]
Csb	1.17	[0.18–7.63]		1.93	[0.71–5.27]		0.51	[0.10–2.65]
Crum	0.68	[0.13–3.46]		2.56	[1.00-6.56] ^*^		0.49	[0.11–2.09]
Ccat	0.38	[0.07–1.99]		0.39	[0.15–1.03]		1.99	[0.48–8.21]
Cbo	1.47	[0.33–6.54]		2.78	[1.12–6.91] ^*^		0.88	[0.24–3.28]
MAA	0.24	[0.06–0.89] ^*^		0.55	[0.27–1.12]		0.28	[0.09–0.84] ^*^
PR	0.19	[0.02–2.11]		0.51	[0.14–1.83]		0.68	[0.09–4.88]
Ips	4.07	[0.3-55.85]		3.54	[0.87–14.46]		0.59	[0.07–5.36]
CSav	1.45	[0.24–8.68]		1.18	[0.44–3.20]		1.56	[0.35–6.95]
CSfa	1.44	[0.28–7.35]		1.37	[0.54–3.52]		0.69	[0.17–2.78]
CSsb	0.52	[0.12–2.18]		2.15	[0.96–4.79]		0.42	[0.13–1.37]
CSsh	1.41	[0.38–5.26]		0.56	[0.27–1.15]		1.01	[0.34–2.96]
CSra	0.76	[0.14–4.14]		0.61	[0.22–1.68]		0.75	[0.18–3.18]
CSps	0.76	[0.17–3.35]		0.53	[0.22–1.25]		2.57	[0.75–8.75]
Fat	0.46	[0.03–6.16]		0.39	[0.08–1.80]		3.11	[0.35–27.29]
Fap	2.15	[0.14–33.35]		0.32	[0.05–2.16]		49.70	[4.5-548.93] ^***^
Fo	2.67	[0.26–27.9]		0.59	[0.13–2.67]		0.79	[0.11–5.78]
Ffs	3.14	[0.39–25.31]		2.10	[0.58–7.52]		0.38	[0.06–2.37]
Frs	0.19	[0.01–2.76]		1.16	[0.22–6.22]		0.10	[0.01–0.95] ^*^
Fop	0.39	[0.04–3.49]		1.01	[0.29–3.50]		2.49	[0.43–14.45]
Mat	1.74	[0.13–23.51]		1.58	[0.33–7.57]		0.51	[0.06–4.43]
Map	0.63	[0.04–9.13]		0.77	[0.13–4.56]		0.20	[0.02–2.43]
Mo	3.47	[0.35–34.56]		0.68	[0.15–2.95]		2.95	[0.43–20.10]
Mfs	0.65	[0.08–4.99]		0.72	[0.21–2.48]		1.05	[0.18–6.08]
Mrs	8.89	[0.70-112.82]		1.50	[0.26–8.63]		0.64	[0.06–6.85]
CTea	0.26	[0.03–2.29]		32.40	[7.53-139.11] ^***^		1.64	[0.27–9.96]
CTpa	1.43	[0.13–16.26]		0.51	[0.08–3.03]		1.22	[0.15–10.13]
CTsa	5.55	[0.58–53.34]		0.27	[0.05–1.40]		0.43	[0.05–3.60]
CTen	5.54	[1.43–21.44] ^*^		1.15	[0.52–2.52]		0.40	[0.11–1.37]
CTpn	0.58	[0.10–3.23]		0.50	[0.19–1.34]		3.78	[0.89–16.03]
NSSI (ref: 0)	——	——		2.68	[1.51–4.76] ^***^		3.58	[2.06–6.21] ^***^
SI (ref: 0)	3.21	[1.79–5.73] ^***^		——	——		19.90	[11.45–34.64] ^***^
SA (ref: 0)	3.07	[1.76–5.38] ^***^		18.90	[10.97–32.69] ^***^		——	——

^*^p < 0.05, ^**^p < 0.01, ^***^p < 0.001; *OR* = odds ratios; *95%CI* = 95% confidence interval.

## Discussion

In our study, we gained insight into the network structure of the associating factors of self-injuries in a student population. The factors that positively related to NSSI, broadly involving proximal, mediating, and distal, while those connecting with suicidality are focused on proximal. Emotional trauma pertaining to paternal parenting is associated with self-injuries.

The mediating factors, centering on emotion dysregulation, had more extensive effects on NSSI than suicidality. Emotion-regulatory processes were categorized into stages (identification, selection, and implementation) [30], failure at any of which could send individuals plunging into emotional dysregulation [30, 31]. Acceptance, often considered a theoretically more “adaptive” strategy, is generally assumed to either have no association with or negatively correlate with maladaptive behaviors [32]. However, in our study, the acceptance strategy exhibited a positive association with NSSI, representing the strongest connection within the self-injury network and NSSI shortest path, as well as yielding the highest OR in the regression analysis. Prior research has identified that using acceptance strategy only may reflect disengagement as a form of resignation, potentially hindering optimal outcomes in stressful or challenging environments [33, 34]. For instance, acceptance may be linked to a negative self-concept (e.g., helplessness), where individuals passively endure distress without the ability to alter their circumstances. In such cases, acceptance may serve as an avoidance strategy, with individuals internalize pain rather than actively processing and resolving it, potentially manifesting in NSSI as a coping mechanism. However, when accompanied by some form of flexible adjustment, the situation may change. The Dual-Process Model proposed an integrated construct in which individuals, on the one hand, modify self-processes or interpret events to align with the outcome, while on the other hand, they accept the environment as it remains unchanged [34]. In our study, positive refocusing strategy was also identified associated with NSSI in the self-injury network. Positive refocusing involves a cognitive adjustment, directing attention toward pleasant thoughts rather than the negative event [23]. Therefore, we considered that combing the acceptance strategy with appropriate alternative adjustments (e.g., positive refocusing) may enhance coping flexibility and potentially reduce the occurrence of NSSI.

Experiencing trauma is significantly associated with self-injury. Growing environment is generally regarded as a distal factor for self-injuries among adolescents, but emotional neglect itself could be a vulnerability to NSSI instead of being interfered in other processes. In our network, distal factors seemed to connect with other factors across three primary pairs of interactions: *DepA*-*CTpn*, *BorS*-*Fo*, and *NSSI*-*CTen*. This implies that depressive mood, borderline symptoms, and NSSI bridge the gap between past and present. Repairing the effects of being neglected in childhood and being over-intervened from father may be helpful to the improvement of NSSI, even to current clinical symptoms. Of note, such interpretations are based on direct observation of the graph and should be interpreted with caution until they can be verified using statistics. Similarly, our study also identified the impact of childhood trauma and paternal parenting on suicidality. Experiencing emotional abuse in childhood had the highest OR for SI, consistent with the previous study [35]. Recent study has further demonstrated that the bidirectional relationship between emotional abuse and SI perpetuates a harmful cycle [35]. Notably, the father’s abusive and punitive parenting style exhibited the highest OR for SA. Experiencing any form of violence during childhood increased the odds of SA [36]. Interestingly, although both fathers and mothers may adopt abusive and punitive parenting styles, maternal use of such methods did not show a significant association with SA. This may reflect the unique role of the father in impacting an individual’s psychological health. Previous research has also demonstrated that fathers’ parenting styles have compensatory effects in alleviating adolescent mental problems [7]. Therefore, clinical interventions aimed at repairing the father-child relationship may facilitate trauma recovery and serve as key targets for breaking the vicious cycle of psychiatric or psychological problems, highlighting the need for further research into the specific impact of paternal behavior on mental health outcomes.

Aside from the environment influences, proximal factors, related to mental disorder symptoms, exhibit the strongest association with suicidality. A study investigated the trends in suicidality, about 80.5–82.0% met the criteria for one or more mental disorders; anxiety disorders were the most common class of disorders among subjects with SI (60.6–62.8%) and SA (70.4–70.9%), and MDD was the most common individual disorder among SI (38.9–41.9%) [37]. We found a particularly strong correlation in our study between anxiety symptoms and suicidality, but the impact of depressive symptoms was lower than expected. When it measured as a continuous clinical or psychological variable, depression could distinguish those who attempt suicide from those who simply have SI (without SA), while depression diagnosis did not [38]. That could affect the results. Additionally, we found that PD symptoms significantly interacted with suicidality in the network analysis. As mentioned above, SA possessed a relatively high specificity for borderline PD, perhaps due to the disorder’s characteristic emotional instability [39]. The network analysis also displayed a direct association between avoidant PD symptoms and SI, but their mechanism was not clear. Although we were unable to verify whether the individuals in our study met actual diagnostic criteria, symptoms of mental disorders seem to affect suicidal tendencies.

Additionally, a closer connection was found between dispositional mindfulness and self-injuries, manifesting more direct effects on SIBs (including NSSI and SA). A previous study has shown that dispositional mindfulness plays a key role in lowering mood disturbance/stress and fosters self-regulation that perhaps moderates the occurrence of extreme thoughts or dangerous behavior. Therefore, to prevent individuals from engaging in SIBs, we can help the students to cultivate and train mindfulness awareness abilities.

Several limitations of our study should be recognized. One concern is that our participants came from non-clinical samples, and it is unknown whether the self-reporting symptoms of these students reached the diagnostic criteria for mental disorders. As well, we lacked objective indicators to support the results, and instead relied on purely subjective reporting scales. Future studies from non-clinical samples should determine whether the measured symptoms met the disorder criteria, and supplement objective materials to further verify the results. Furthermore, due to the cross-sectional design, the causation between associating factors and self-injuries cannot be substantiated. Future longitudinal studies are needed to explore the dynamic process and interaction mechanism.

The current study attempted to interpret the interactions between multidimensional factors and types of self-injury in students. To better understand the extreme ideations and dangerous behaviors, the network structure was utilized as it provides a great deal of evidence from a global perspective. Relying exclusively on acceptance strategy may constitute an avoidance pattern, impeding the ability to confront emotional distress. Cultivating mindfulness attention awareness and applying acceptance strategy in a dialectical manner, such as in conjunction with other adjustable strategies, may prove beneficial in reducing NSSI. Emotional trauma experiencing in childhood (particularly paternal abuse and punishment) may be responsible for provoking and intensifying suicidality. Clinical intervention aimed at repairing the father-child relationship may be helpful to recover from emotional trauma and improve current symptoms and self-injuries.

## Electronic supplementary material

Below is the link to the electronic supplementary material.

Supplementary Material 1

## Data Availability

The datasets generated and/or analyzed during the current study are not publicly available due to privacy and ethical restrictions but are available from the corresponding author upon reasonable request.
