# Supplementary material for: Interactions of factors in self-injuries among enrolled students: a network approach
Source: Ann Gen Psychiatry. 2025 May 16;24:28. doi: 10.1186/s12991-025-00570-0 (PMC12084963; doi:10.1186/s12991-025-00570-0)
Supplement: Supplementary file 1 — Supplementary Material 1 [file 12991_2025_570_MOESM1_ESM.docx]

**Interactions of Factors in Self-injuries among Enrolled Students: A Network Approach**

Table S1 displays the description of variables for the network structure including respective recording, scoring and abbreviations.

| **Table S1** Detailed description of variables for the network structure (China, 2020-2021). | | | | |
| --- | --- | --- | --- | --- |
| **Node** | **Abbreviation** | **Meaning in the network** | **Scale in questionnaire** | **Scale in network** |
| 1 | *DepA* | depressive affect | SDS; ordinal (4-point scale, 20 items) | binary (1= ScalSco greater than or equals to 53; 0= ScalSco fewer than 53) |
| 2 | *ManA* | manic or hypomanic affect | MDQ; binary (13 items) | binary (1= TotSco greater than or equals to 7; 0= TotSco fewer than 7) |
| 3 | *AnxA* | anxiety affect | SAS; ordinal (4-point scale, 20 items) | binary (1= ScalSco greater than or equals to 50; 0= ScalSco fewer than 50) |
| 4 | *BorS* | borderline symptom | BSL-23; ordinal (5-point scale, 23 items) | binary (1= AVG greater than or equals to 2.8; 0= AVG fewer than 2.8) |
| 5 | *PPD* | paranoid PD | PDQ-4; binary (7 items) | binary (1= TotSco greater than or equals to 4; 0= TotSco fewer than 4) |
| 6 | *ScPD* | schizoid PD | PDQ-4; binary (7 items) | binary (1= TotSco greater than or equals to 4; 0= TotSco fewer than 4) |
| 7 | *SctPD* | schizotypal PD | PDQ-4; binary (9 items) | binary (1= TotSco greater than or equals to 5; 0= TotSco fewer than 5) |
| 8 | *AnPD* | antisocial PD | PDQ-4; binary (8 items) | binary (1= TotSco greater than or equals to 3; 0= TotSco fewer than 3) |
| 9 | *BPD* | borderline PD | PDQ-4; binary (9 items) | binary (1= TotSco greater than or equals to 5; 0= TotSco fewer than 5) |
| 10 | *HPD* | histrionic PD | PDQ-4; binary (8 items) | binary (1= TotSco greater than or equals to 5; 0= TotSco fewer than 5) |
| 11 | *NPD* | narcissistic PD | PDQ-4; binary (9 items) | binary (1= TotSco greater than or equals to 5; 0= TotSco fewer than 5) |
| 12 | *AvPD* | avoidant PD | PDQ-4; binary (7 items) | binary (1= TotSco greater than or equals to 4; 0= TotSco fewer than 4) |
| 13 | *DPD* | dependent PD | PDQ-4; binary (8 items) | binary (1= TotSco greater than or equals to 5; 0= TotSco fewer than 5) |
| 14 | *OPD* | obsessive-compulsive PD | PDQ-4; binary (8 items) | binary (1= TotSco greater than or equals to 4; 0= TotSco fewer than 4) |
| 15 | *EI* | emotion intelligence | WLEIS; ordinal (7-point scale, 16 items) | continuous (TotSco); higher emotional intelligence leads to higher score. |
| 16 | *Alx* | severity of alexithymia | TAS; ordinal (5-point scale, 26 items) | continuous (TotSco); the more severe the alexithymia, the higher the score. |
| 17 | *Cprf* | positive refocusing strategy | CERQ; ordinal (5-point scale, 4 items) | continuous (TotSco);  the score indicates one's tendency thought after experiencing threatening or stressful life events. |
| 18 | *Crop* | refocus on planning strategy | CERQ; ordinal (5-point scale, 4 items) |  |
| 19 | *Cpra* | positive reappraisal strategy | CERQ; ordinal (5-point scale, 4 items) |  |
| 20 | *Cacc* | acceptance strategy | CERQ; ordinal (5-point scale, 4 items) |  |
| 21 | *Cpip* | putting into perspective strategy | CERQ; ordinal (5-point scale, 4 items) |  |
| 22 | *Csb* | self-blame strategy | CERQ; ordinal (5-point scale, 4 items) |  |
| 23 | *Crum* | rumination strategy | CERQ; ordinal (5-point scale, 4 items) |  |
| 24 | *Ccat* | catastrophizing strategy | CERQ; ordinal (5-point scale, 4 items) |  |
| 25 | *Cbo* | blaming others strategy | CERQ; ordinal (5-point scale, 4 items) |  |
| 26 | *MAA* | mindful attention awareness | MAAS; ordinal (6-point scale, 15 items) | continuous (AVG); higher score reflects higher level of dispositional mindfulness. |
| 27 | *PR* | psychological resilience | CD-RISC; ordinal (5-point scale, 25 items) | continuous (AVG); the greater resilience, the higher the score. |
| 28 | *Ips* | impulsive trait | BIS; ordinal (5-point scale, 30 items) | continuous (ScalSco); a high score indicates a single common latent trait of impulsivity. |
| 29 | *CSav* | avoiding | CSQ; ordinal (4-point scale, 11 items) | continuous (AVG); a high score indicates one's preference coping style under stress. |
| 30 | *CSfa* | fantasizing | CSQ; ordinal (4-point scale, 10 items) |  |
| 31 | *CSsb* | self-blaming | CSQ; ordinal (4-point scale, 10 items) |  |
| 32 | *CSsh* | seeking help | CSQ; ordinal (4-point scale, 10 items) |  |
| 33 | *CSra* | rationalizing | CSQ; ordinal (4-point scale, 11 items) |  |
| 34 | *CSps* | problem solving | CSQ; ordinal (4-point scale, 12 items) |  |
| 35 | *Fat* | father affectionate & tolerant | C-EMBU; ordinal (4-point scale, 19 items) | continuous (TotSco);  the score reflects the degree to which each parent was affectionate and tolerant, abusive and punitive, overinvolved, rejecting and shaming, overprotective, and favored the subject. |
| 36 | *Fap* | father abusive & punitive | C-EMBU; ordinal (4-point scale, 12 items) |  |
| 37 | *Fo* | father overinvolved | C-EMBU; ordinal (4-point scale, 10 items) |  |
| 38 | *Ffs* | father favored subject | C-EMBU; ordinal (4-point scale, 5 items) |  |
| 39 | *Frs* | father rejecting & shaming | C-EMBU; ordinal (4-point scale, 6 items) |  |
| 40 | *Fop* | father overprotective | C-EMBU; ordinal (4-point scale, 6 items) |  |
| 41 | *Mat* | mother affectionate & tolerant | C-EMBU; ordinal (4-point scale, 19 items) |  |
| 42 | *Map* | mother abusive & punitive | C-EMBU; ordinal (4-point scale, 9 items) |  |
| 43 | *Mo* | mother overinvolved | C-EMBU; ordinal (4-point scale, 16 items) |  |
| 44 | *Mfs* | mother favored subject | C-EMBU; ordinal (4-point scale, 5 items) |  |
| 45 | *Mrs* | mother rejecting & shaming | C-EMBU; ordinal (4-point scale, 8 items) |  |
| 46 | *CTea* | emotional abuse in childhood | CTQ-SF; ordinal (5-point scale, 5 items) | continuous (TotSco); the score indicates self-reported experiences of emotional, physical and sexual abuse, and emotional and physical neglect in childhood. |
| 47 | *CTpa* | physical abuse in childhood | CTQ-SF; ordinal (5-point scale, 5 items) |  |
| 48 | *CTsa* | sexual abuse in childhood | CTQ-SF; ordinal (5-point scale, 5 items) |  |
| 49 | *CTen* | emotional neglect in childhood | CTQ-SF; ordinal (5-point scale, 5 items) |  |
| 50 | *CTpn* | physical neglect in childhood | CTQ-SF; ordinal (5-point scale, 5 items) |  |
| 51 | *NSSI* | non-suicidal self-injury | OSI; question | binary (1=TotSco greater than 0; 0=TotSco equals to 0) |
| 52 | *SI* | suicidal ideation | C-SSRS; binary (5 items) | binary (1=TotSco greater than 0; 0=TotSco equals to 0) |
| 53 | *SA* | suicide attempt | C-SSRS; binary (4 items) | binary (1=TotSco greater than 0; 0=TotSco equals to 0) |
| PD= personality disorder; CER= cognitive emotion regulation; CS= coping style; CT= childhood trauma;  SDS/SAS= Self-rating Depression/Anxiety Scale; MDQ= Mood Disorder Questionnaire; BSL-23= the Short Version of the Borderline Symptom List; PDQ-4= Personality Diagnostic Questionnaire 4th edition; WLEIS= Wong-Law Emotional Intelligence Scale; TAS= Toronto Alexithymia Scale; CERQ= Cognitive Emotion Regulation Questionnaire; MAAS= Mindfulness Attention Awareness Scale; CD-RISC= Connor-Davidson Resilience Scale; BIS= Barratt Impulsiveness Scale; CSQ= Coping Style Questionnaire; C-EMBU= Chinese version of the Swedish EMBU inventory; CTQ-SF= Childhood Trauma Questionnaire 28-item short form; OSI= Ottawa/Queen’s Self-Injury Questionnaire; C-SSRS= Columbia Suicide Severity Rating Scale (C-SSRS);  TotSco= total score; AVG= average score; ScalSco= scaled score;  Associating factors= node 1-50 (proximal factors= node 1-14, mediating factors= node 15-34, distal factors= node 35-50); suicidal and non-suicidal self-injuries= node 51-53. | | | | |

Fig. S1 presents the accuracy and stability of the network and includes bootstrap samples for the factor network (Fig. S1 A) and self-injury network (Fig. S1B).

| 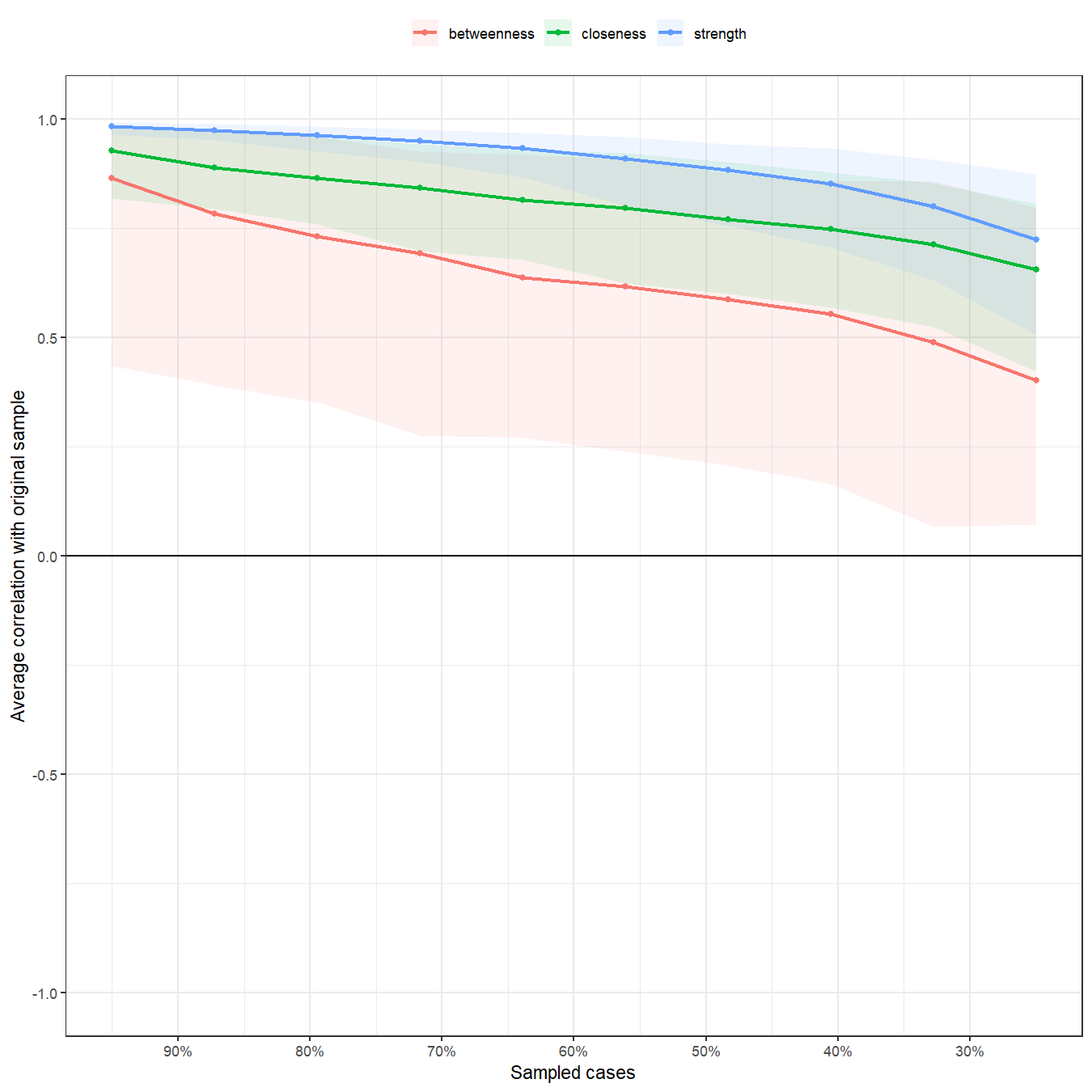 | 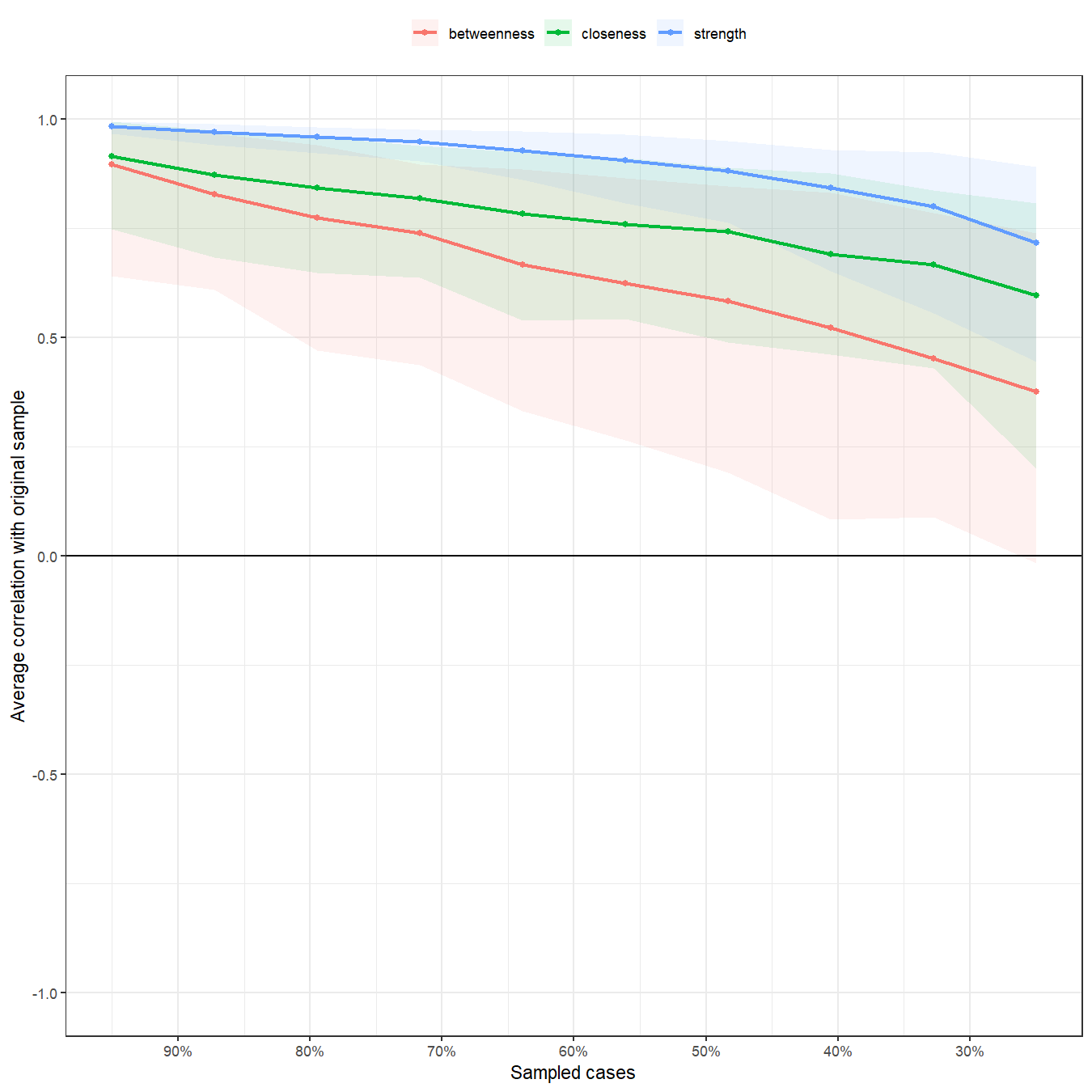 |
| --- | --- |
| **A. Factor Network** | **B. Self-injury Network** |
| **Fig. S1** The stability of centrality metrics (strength, closeness, betweenness) by estimating network models based on case-dropping bootstrap for A. Factor Network (associating factors) and B. Self-injury Network (associating factors with suicidal and non-suicidal self-injuries). Lines indicate the means, and areas indicate the range from 2.5^th^ to 97.5^th^ quantile. Strength centralities are depicted with blue lines and areas, closeness centralities are shown in green, and betweenness centralities are shown in red (China, 2020-2021). | |
